# Clinical study of anti-snake venom blockade in the treatment of local tissue necrosis caused by Chinese cobra *(Naja atra)* bites

**DOI:** 10.1371/journal.pntd.0010997

**Published:** 2022-12-16

**Authors:** Linsheng Zeng, Jingjing Hou, Cuihong Ge, Yanjun Li, Jianhua Gao, Congcong Zhang, Peiying Huang, Jiayu Du, Zhizhun Mo, Yuxiang Liu, Zhongyi Zeng

**Affiliations:** 1 Department of Emergency, Shenzhen Traditional Chinese Medicine Hospital, Shenzhen, China; 2 Guangzhou University of Chinese Medicine, Guangzhou Panyu Central Hospital,Guangzhou,China; 3 The Second Clinical Medical School of Guangzhou University of Chinese Medicine, Guangzhou, China; Liverpool School of Tropical Medicine, UNITED KINGDOM

## Abstract

**Objective:**

This study aimed to evaluate the clinical therapeutic efficacy of anti-snake venom serum blockade in treating local tissue necrosis caused by Chinese cobra (*Naja atra)* bites.

**Methods:**

Patients bitten by a Chinese cobra (*Naja atra)* (n = 50) that met the inclusion criteria were randomly divided into two groups: the experimental group (n = 25) and the control group (n = 25). The experimental group received regular as well as anti-snake venom serum blocking treatment, whereas regular treatment plus chymotrypsin blocking therapy was given to the control group. The necrotic volumes around snake wounds in these groups were detected on the first, third and seventh days. On the third day of treatment, some local tissues in the wounds were randomly selected for pathological biopsy, and the necrosis volume of the local tissue was observed. Furthermore, the amount of time required for wound healing was recorded.

**Results:**

On the third and seventh days post-treatment, the necrotic volume of the wound of the experimental group was much smaller than that of the control group, and the experimental group’s wound healing time was shorter than that of the control group (all *p* < *0.05*). Moreover, the pathological biopsies taken from the control group showed nuclear pyknosis, fragmentation, sparse nuclear density, and blurred edges, and the degree of necrosis was much higher than that of the experimental group.

**Conclusions:**

Anti-snake venom blocking therapy is a new and improved therapy with good clinical effect on local tissue necrosis caused by Chinese cobra bites; moreover, it is superior to conventional chymotrypsin blocking therapy in the treatment of cobra bites. It can better neutralize and prevent the spread of the toxin, reduce tissue necrosis, and shorten the course of the disease by promoting healing of the wound. Furthermore, this treatment plan is also applicable to wound necrosis caused by other snake toxins, such as tissue necrosis caused by elapidae and viper families.

**Clinical Trial Registration:**

This trial is registered in the Chinese Clinical Trial Registry, a primary registry of International Clinical Trial Registry Platform, World Health Organization (Registration No. ChiCTR2200059070; trial URL:http://www.chictr.org.cn/edit.aspx?pid=134353&htm=4).

## Author summary

Chinese cobra bites often cause local tissue necrosis and lead to physical disability, which is the difficulty of treatment. Considering that the cytotoxin in cobra is the main pathogenic factor causing local tissue necrosis, we proposed injecting anti-snake venom locally to increase its concentration in the wound, so as to neutralize the cytolytic activity of snake venom. In order to verify the clinical efficacy of the therapy, a randomized controlled trial were conducted: the experimental group was treated with anti-snake venom blocking therapy, while the control group was treated with chymotrypsin blocking therapy. The clinical efficacy was evaluated by comparing the local wound necrosis, wound healing time, etc. as the indicators. Then, the results showed that the experimental group had smaller wound necrosis area and also much shorter healing time than the control group, which clearly indicated that the local blocking treatment with anti-snake venom serum has much better clinical efficacy. Meanwhile, theoretically, the anti-snake venom blocking therapy is also suitable for the treatment against other snake bites. So, in future we expect to conduct more clinical studies on this, which will provide information on new treatments with much better clinical efficacy for patients.

## 1. Introduction

Snakebite envenoming was added to the list of neglected tropical diseases in 2017 by the World Health Organization [1], and Chinese cobras are widely distributed around the world, posing a great threat to human safety. Therefore, we must refocus on the treatment and research of snakebites. Chinese cobras, one of the top ten venomous snakes in China are mainly distributed in provinces that are south of the Yangtze River, including Taiwan, Hainan, Hong Kong, and Macao [2].

The toxins secreted by Chinese cobra include neurotoxins, hematotoxins, and cytotoxins, which are classified according to clinical symptoms [3,4]. Neurotoxins can block neuromuscular conduction, which can lead to muscle paralysis and respiratory failure in severe cases [5,6]. Cobrotoxin is the main neurotoxic protein isolated from the venom of the Chinese cobra (*Naja atra*) [7]. Hematotoxicity destroys the function of the blood system [8]. The main phospholipase A_2_ and its isozymes inhibit blood coagulation by different mechanisms [9,10]. Cytotoxins directly damage skin tissues, manifesting as severe swelling of tissue cells around the necrotic volume, vacuolar degeneration, microvascular embolism [4,11], and other damaging changes, followed by the development of extensive necrosis, with wounds that are often difficult to heal over time, and in severe cases, they cause limb disability or functional impairment [12].

Cytotoxins have been found to be the main components of Chinese cobra venom, accounting for more than half (54.2%) of the venom [13].A variety of cytotoxins analogs have been discovered that act primarily by disrupting the bilayer membrane of cells [14]. The toxin composition varies by species, with the venom of the Chinese cobra being more abundant in cytotoxins compared to that of *Naja kaouthia* and *Naja siamensis* [15]. Clinical observations have shown that intravenous anti-snake venom treatment is less effective in detoxifying local tissue necrosis caused by cobra bites [13,16]. The main reason may be that after intravenous injection anti-snake venom, it needs to pass through the body via the bloodstream to reach the local wound. As a result, local wounds will retain a high concentrations of toxins [17], the local tissue continues to ulcerate under the action of cytotoxin.

Generally, Chinese cobra bites are associated with disability, and often cause local tissue necrosis [18]. Despite early administration of intravenous anti-snake venom (< 6 hours), more than half of all cobra bites developed local tissue necrosis, necessitating debridement or other surgical intervention [19], which is the primary difficulty faced in this treatment. Therefore, in recent years, new treatments have been proposed to treat local tissue necrosis caused by snake venom. Chymotrypsin (EC 3.4.21.1) blocking therapy is a kind of regular treatment plan against Chinese cobra bite [20]. Generally, cobra cytotoxins is a kind of small three-looped proteins composed of approximately 60 amino acid residues [14], while chymotrypsin (EC 3.4.21.1) is a proteolytic enzyme with endopeptidase function. Therefore, chymotrypsin has certain degradation effect on snake venom. Furthermore, chymotrypsin which has been used in clinics since 1960, can effectively promote the faster recovery of acute tissue injury by removing necrotic tissue proteins [21], and is conducive to wound healing. However, chymotrypsin blocking therapy has never solved the problem of wound necrosis caused by snake venom. Considering that cytotoxin is the main pathogenic factor causing local tissue necrosis, neutralization or inactivation of the same is becoming the key to its treatments.

Thus, in this study, we proposed to adopt the method of local anti-snake venom injection to strengthen the anti-snake venom effect, which aims to neutralize the cytolytic activity of snake venom through high concentration of anti-snake venom. Thereby, this study sets two treatment schemes: the chymotrypsin blocking treatment scheme and anti-snake venom blocking treatment scheme. Both of them are combination therapies that adopt intravenous injection of anti-snake venom as basic treatment; while their difference is that the local treatment uses either chymotrypsin enzyme (control group) or anti-snake venom (experimental group). The whole therapeutic schedule of this study is shown in the S1 Fig. Here the emphasis is on the fact that the anti-snake venom blocking treatment scheme aims to neutralize the cytolysis of the toxin through intravenous and local injection. Based on this hypothesis, a single-anonymous, parallel groups, randomized controlled trial was designed and conducted to compare the clinical efficacy of these two treatment regimens.

## 2. Materials and methods

### 2.1 Ethics statement

This trial was conducted according to the Declaration of Helsinki. Before all the patients were enrolled in the study, the researcher gave full information, which mainly included the purpose and content of the study and the risks and benefits of participating in the study. All the patients received and signed the written informed consent. This study was reviewed and approved by the Ethics Committee of Shenzhen Traditional Chinese Medicine Hospital with approval number K2020-023-01.

### 2.2 Trial design

#### 2.2.1 Participants recruitment

1) Randomization and randomization concealment

The researcher adopted the EXCEL (Microsoft office 2017) random number table method to generate the random sequence. To conceal the random sequence for the researchers, an opaque sealed envelope numbered sequentially was shuffled. After the volunteer participants agreed to join and signed the informed consent form, they received the envelope and returned it to the researcher, who assigned the participant to the experimental group or the control group according to the randomization list.

2) Process of participation in the study

**Fig 1:**
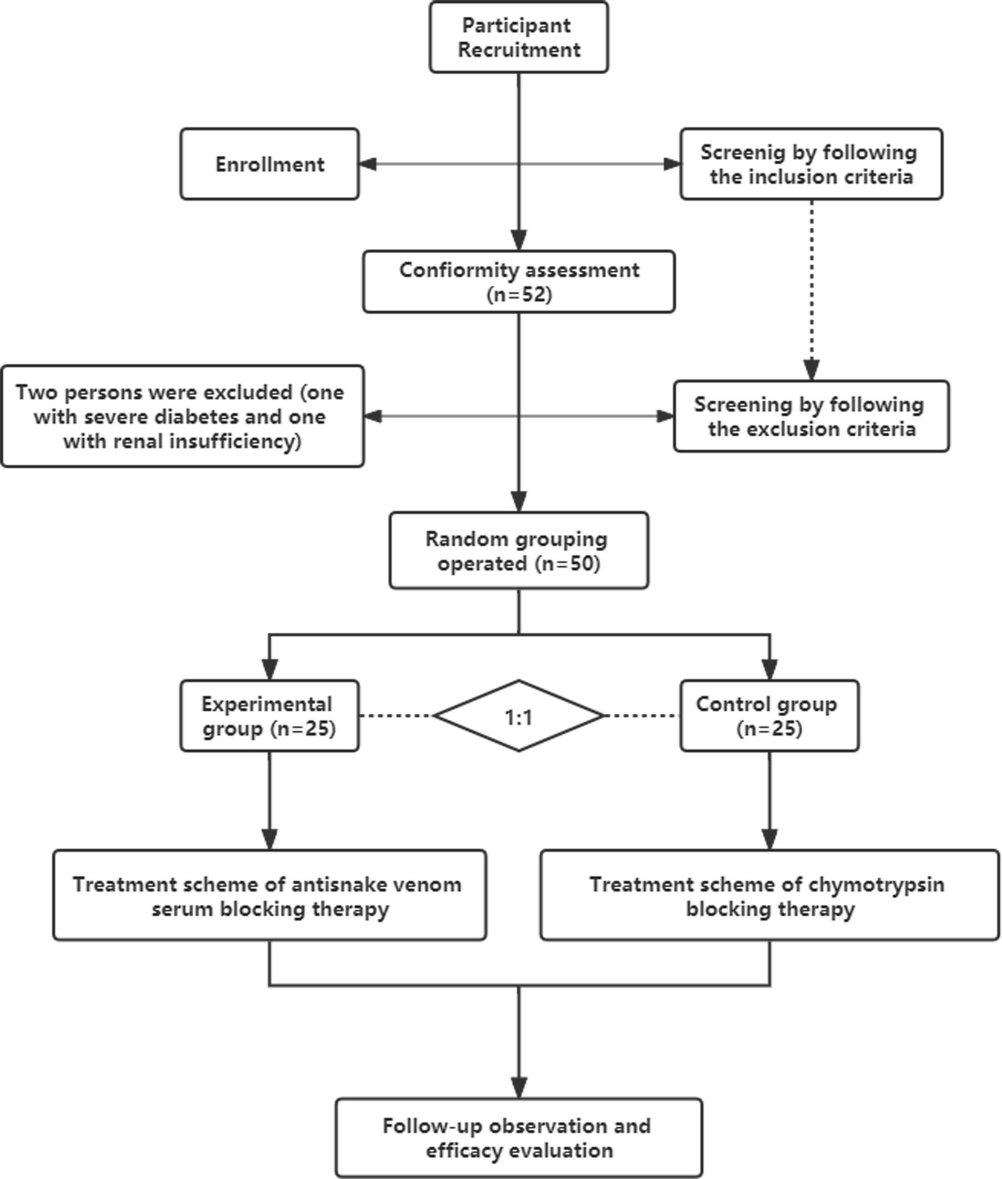
Flow diagram of patient enrollment treatment and follow-up.

This study is a single-anonymous, parallel groups, randomized controlled trial. Shown in Fig 1, the research selection process was strictly in accordance with the inclusion criteria and exclusion criteria. Chinese cobra (*Naja atra*) bite patients enter the screening process after information registration. The study cases were from the emergency department of Shenzhen Traditional Chinese Medicine Hospital from July 9, 2020 to July 8, 2021: a total of 52 cases met the inclusion criteria, including one with severe diabetes and one with renal insufficiency. Based on the exclusion criteria, two (one with severe diabetes and one with renal insufficiency) were excluded from the study, and finally 50 cases were included in the randomized grouping. Then according to the 1:1 ratio, all these cases were divided into the experimental group and the control group. The experimental group was treated with anti-snake venom blocking therapy, while the control group was treated with chymotrypsin blocking therapy. Finally, the patients were followed up until the wound healed, and the efficacy was evaluated.

#### 2.2.2 Diagnostic criteria

According to the 2018 Chinese Expert Consensus on Snakebite Treatment [22] and the relevant content of the diagnosis and treatment protocol of Shenzhen Traditional Chinese Medicine Hospital, the cobra bite cases were diagnosed and confirmed based on the following criteria:

1. A history of snakebites.
2. The patient saw that it was a Chinese cobra, the described appearance of the snake matched the characteristics of a Chinese cobra, or a medical personnel identified the snake as a cobra.
3. The wound showed bite marks, local redness, and pain. Moreover, the wound appeared blackened with possible early signs of gangrene.
4. Systemic neuromuscular paralysis symptoms: symptoms appear mostly 1–6 hours after the bite; in mild cases, dizziness, chest tightness, weakness of the limbs; and in severe cases, slurred speech, blurred vision, drooping eyelids, salivation at the corners of the mouth, closed teeth, difficulty swallowing, weak and shallow breathing or difficulty breathing, cyanosis, etc. The main symptoms of blood system damage are: blood stasis spots present all over the body; blood in the urine; and even hematemesis, hemoptysis, hematochezia, or bleeding; followed by anemia, severe shock, etc. The diagnosis was made based on the presence of symptoms (1), (2), and (3) or (1), (2), and (4).

#### 2.2.3 Inclusion criteria

1. The patient met the diagnostic criteria above, and the wound showed local tissue necrosis.
2. Age ≥ 18 years old and ≤ 60 years old.
3. Patients did not participate in other trials or treatments before inclusion.

#### 2.2.4 Exclusion criteria

(a) Pregnant and lactating women;
(b) Patients with various psychiatric diseases, confusion, or dementia;
(c) Patients with varicose veins or edema in the affected limbs;
(d) Patients with diabetes mellitus and long-term poor glycemic control combined with the diabetic foot;
(e) Patients with allergies to chymotrypsin or anti-cobra venom serum;
(f) Patients with cardiac, hepatic, or renal insufficiency;
(g) Patients with combined tumors, hematologic diseases, or immunodeficiency;
(h) Patients treated with glucocorticoids for a long time before admission.

#### 2.2.5 Evaluation of sample size

The sample size was estimated according to the main efficacy evaluation indicators, and the necrosis volume of the snake bite wound (mm^3^) is set as the main efficacy evaluation indicator. Referring to the relevant literature and previous clinical observations, it is found to be significant when the necrosis volume of difference between the wound and healthy volume is up to at least 23 within the same sample size. We selected MedSci online calculator to estimate the samples size and get the parameters; here the mean of the test group is about 37, the standard deviation (SD) is about 15, the mean of the control group is about 60, the SD is about 30, and α = 0.05, β = 0.10 (power = 0.90), β takes a two-sided value, and the maximum possibility of quitting midway is 20%. This estimation tool of MedSci yielded 50 cases, with N = 25 as the total number.

#### 2.2.6 Single-anonymous setup

The researchers who administered the treatment were not anonymized, but the patients and the measurers were, and the researchers who implemented the closed therapy were separated from the outcome measurers to reduce bias and ensure that the results of the experiment were objective and true.

#### 2.2.7 Case dropout and trial termination

All participants who passed the screening and entered the trial, were considered as dropout cases if they were not able to complete the observation specified in the protocol regardless of when and why they withdrew. In the course of the trial, once serious adverse events occur, the trial would be immediately terminated and treated as case dropout. For those who quit the trial midway, take active measures to record the last test result as far as possible.

### 2.3 Treatment methods

#### 2.3.1 Conventional therapies

According to the routine treatment plan of the Shenzhen Traditional Chinese Medicine Hospital, Department of Snakebite, the procedure which is shown in S1 Fig was performed as follows [23]:

(1) *Naja naja (atra)* Antivenin administered by intravenous injection (Shanghai Sailun Biotechnology Co., Ltd., Lot No. 20190701);
(2) Injection of tetanus antitoxin administered (Lanzhou Biological Products Research Institute Co., Ltd., Lot No. 202008031);
(3) Wound repeatedly rinsed with 5‰ potassium permanganate solution (Jinan Kangfusheng Pharmaceutical, Lot No. 210420) or hydrogen peroxide solution (Guangdong Hengjian Pharmaceutical Co., Ltd., Lot No. 200328);
(4) Dexamethasone injection administered (Sinopharm Group Rongsheng Pharmaceutical Co., Ltd., Lot No. 2103220) 10 mg IV daily for 3 days;
(5) Second generation cephalosporins used to prevent and control local infections;
(6) Blood transfusion treatment administered: platelets < 50×10^9^/L, if there was bleeding or platelet < 5×10^9^/L, transfusion of platelets; if fibrinogen < 0.8g/L, transfusion of cold precipitation; if PT or APTT > 1.5 times normal, transfusion of plasma; if red blood cells < 60 g/L or HCT < 20%, transfusion of red blood cells.

#### 2.3.2 Study design of local blocking therapy

(1) The wound and its surrounding 5 cm were disinfected successively with 0.5% povidone-iodine disinfectant solution (Shenzhen Andover Disinfection High-Tech Co., Ltd., Lot No. 21081055) and 75% alcohol (Beihai Guofa Marine Biological Industry Co., Ltd., Lot No. 210142) and then rinsed 3 times with saline.
(2) A 10 ml syringe was used with 10 ml of closure solution, and after subcutaneous needle entry, the closure fluid was injected into the wound.
(3) With the wound as the center, a circular injection of closure solution was done around the wound at 1–2 cm to infiltrate the volume. Table 1 shows the injection dose for different body parts.

**Table 1:** Injection dose for different body parts for local blocking therapy.

Body parts	Injection dose (mL)	
	Open 1–2 cm next to the wound (circular)	Wound site (infiltration injection and irrigation)*
Fingers and toes	2	8
Palm volume	4	6
Sole	4	6
Forearm	5	5
Leg	5	5

* Note: When the wound is closed for treatment, first infiltrate the injection until the local tissue cannot be absorbed, and then rinsed the remaining injection into the wound deeply.

(4) Control group: The closure solution consisted of a mixture of 4000 U of chymotrypsin (Shanghai ShangPharma First Biochemical Pharmaceutical Co., Ltd., Lot No. 2005901), 10 ml of 2% lidocaine solution (Shanghai Chaohui Pharmaceutical Co., Ltd., Approval No. 1812T06) and 5 mg of dexamethasone (Sinopharm Rongsheng Pharmaceutical Co., Ltd., Lot No. 2103220).
(5) Experimental group: The closure solution consisted of a mixture of 1 *Naja naja (atra)* Antivenin (Shanghai Sailun Biotechnology Co., Ltd., Lot No. 20190701, 1000 IU/piece), 10 ml of 2% lidocaine solution (Shanghai Chaohui Pharmaceutical Co., Ltd., Approval No. 1812T06), and 5 mg of dexamethasone (Sinopharm Rongsheng Pharmaceutical Co., Ltd., Lot No. 2103220).
(6) On the first day, local closure was performed once at the time of consultation with the patients.

### 2.4 Assessment index of the response

#### 2.4.1 Measurement of the necrotic volume of the wound

The necrotic volume of the wound is the main therapeutic evaluation index. So, the volume of wound necrosis before closure treatment was measured on the third and seventh days post-treatment. This study set up had measurement specialists and used uniform measurement standards and anonymized measurements (measurement specialists could not obtain patient enrollment information). The three-dimensional volume was measured after debridement and removal of the necrotic tissue: length×width×depth to calculate the measurement of the pressure sore volume. The measurement procedure was as follows: The wound length was detected along the long axis of the body, regardless of the wound’s location on the body. Additionally, the width was measured along the direction perpendicular to the long axis. The depth was measured along the direction perpendicular to the body surface; the widest and longest part of the surface and the deepest depth were measured with the head as the coordinates, the length in the longitudinal direction, the width in the transverse direction, and the depth perpendicular to the body surface. If the wound was irregular, it was measured and predicted with care.

#### 2.4.2 Wound healing time

The time of the cobra bite and the time it took for the wound to heal completely were both recorded, and the time taken for complete wound healing (days) was calculated as follows: Wound healing time (days) = date of complete wound healing—date of cobra bite

#### 2.4.3 Pathological examination of wound tissue

After the third day of the treatment, the wound tissues of these two groups were randomly selected for pathological section detection to further evaluate the severity of local tissue necrosis of the wounds. The testing institution was Department of Pathology in Shenzhen Traditional Chinese Medicine Hospital. Sampling method of wound tissue: after local disinfection of the wound, used 2% lidocaine for local infiltration anesthesia. The tooth mark left by the snake bite was then taken to the center, where 2 mm^3^ tissue was cut from the edge of the tooth mark with a sterile surgical blade and sent to the Department of Pathology for determination.

### 2.5 Statistical methods

SPSS 21.0 software was used to statistically analyze the test data. The measurement data such as age, height, weight, body mass index, time between bite and treatment, snake bite severity score, wound necrosis volume, and wound healing time, were described by the mean ± SD. Moreover, the data conforms to the homogeneity of variance and normal distribution, T-test was adopted for these data analysis. While Sender, Bite site (case), Length distribution of cobras (case) belongs to count data, and the χ2 test was employed for them. Here *p* < *0.05* indicates a statistically significant difference.

## 3. Results

### 3.1 Clinical data analysis

Among the 50 patients, 47 were male and 3 were female. No case dropped out during the study. The youngest and oldest participants were 19 years and 60 years old, respectively. Height ranged from 158 cm to 179 cm, weight ranged from 50 kg to 76 kg, and BMI ranged from 19 to 26. The sites of snakebite envenoming were distributed in the limbs: 22 cases of upper limbs and 28 cases of lower limbs. The shortest period from a venomous snakebite to hospital admission was 10 minutes and the longest was 2 hours. The length range of venomous snakes was from 0 to 3 meters. According to the snakebite severity scale in the 2018 Chinese Expert Consensus on Snakebite Treatment [23], at admission, the lowest score was 2 points, and the highest score was 7 points, with an average score of 3.7 points. There was no significant difference in general data between the two groups of patients (all *p* < *0.05*), and they were comparable, as shown in Table 2.

**Table 2:** Comparison of general data of cobra bite patients between the two groups. (Mean±SD).

Groups		Experimental group	Control group	*χ*^2^/*t*	*P value*
Cases		25	25		
Gender (cases)	Male	24	23	*0*.*355*	*0*.*552*
	Female	1	2		
Age	years	43.16±8.98	45.72±15.18	*0*.*021*	*-0*.*726*
Height	cm	169.4±3.79	170.04±4.61	*0*.*59*	*-0*.*542*
Weight	kg	66.28±5.19	68.44±6.55	*-1*.*292*	*0*.*203*
BMI	-	23.09±1.55	23.62±1.55	*-1*.*215*	*0*.*23*
Bited site (case)	upper extremity	10	12	*0*.*325*	*0*.*569*
	lower limbs	15	13		
Time between bite and treatment(min)		56.84±26.63	47.16±23.10	*0*.*338*	*0*.*564*
Length distribution of cobras (case)	(0,1)meter	6	8	*0*.*463*	*0*.*793*
	(1,2) meter	15	14		
	(2,3)meter	4	3		
Snakebite severity score (points)		3.52±1.12	3.88±1.36	*-1*.*019*	*0*.*435*

### 3.2 Emergency response measures of adverse events

Throughout the study, two patients developed a reaction to the intravenous anti-snake venom, mainly manifested as a sparse rash throughout the body. The following treatment method was immediately provided: the injection speed of anti-cobra venom was reduced from 2 U/min to 0.5U/min; the dose of dexamethasone injection was increased (Sinopharm Rongsheng Pharmaceutical Co., Ltd., batch number: 2103220) with 10mg intravenous drip and any changes in the patient’s symptoms and signs were closely monitored. This procedure ensured that the treatment was not interrupted due to reactions.

### 3.3 Comparison of wound necrosis volume of cobra bite between two study participants groups

From the data shown in Table 3, there was no significant difference in the wound volume between the two groups on the first day of treatment. However, on the third day, the necrotic volume of the wound peaked in both groups. Moreover, the control group (1674.4 ± 972.35 mm^3^) peaked significantly higher than the experimental group (71.16 ± 41.56 mm^3^). Following this, the necrotic volume of the two groups gradually narrowed. However, on the third and seventh days, the necrotic volume of the wound in the experimental group was always significantly smaller than that in the control group (*p* < *0.05*). It can be inferred that the treatment plan for the experimental group was superior to that of the control group since it could more effectively reduce the necrotic volume of the wound.

**Table 3:** Comparison of wound necrosis volume with Cobra bite between two groups of patients. (Mean±SD).

Groups	Cases	Day 1 of treatment(mm^3^)	Day 3 of treatment(mm^3^)	Day 7 of treatment(mm^3^)
Experimental group	25	59.84±36.68	71.16±41.56*	37.28±28.51*
Control group	25	46.20±28.22	1674.44±972.35	172.68±901.06

Note: * compared with the control group (*p* < *0.05*)

### 3.4 Wound healing time

**Fig 2:**
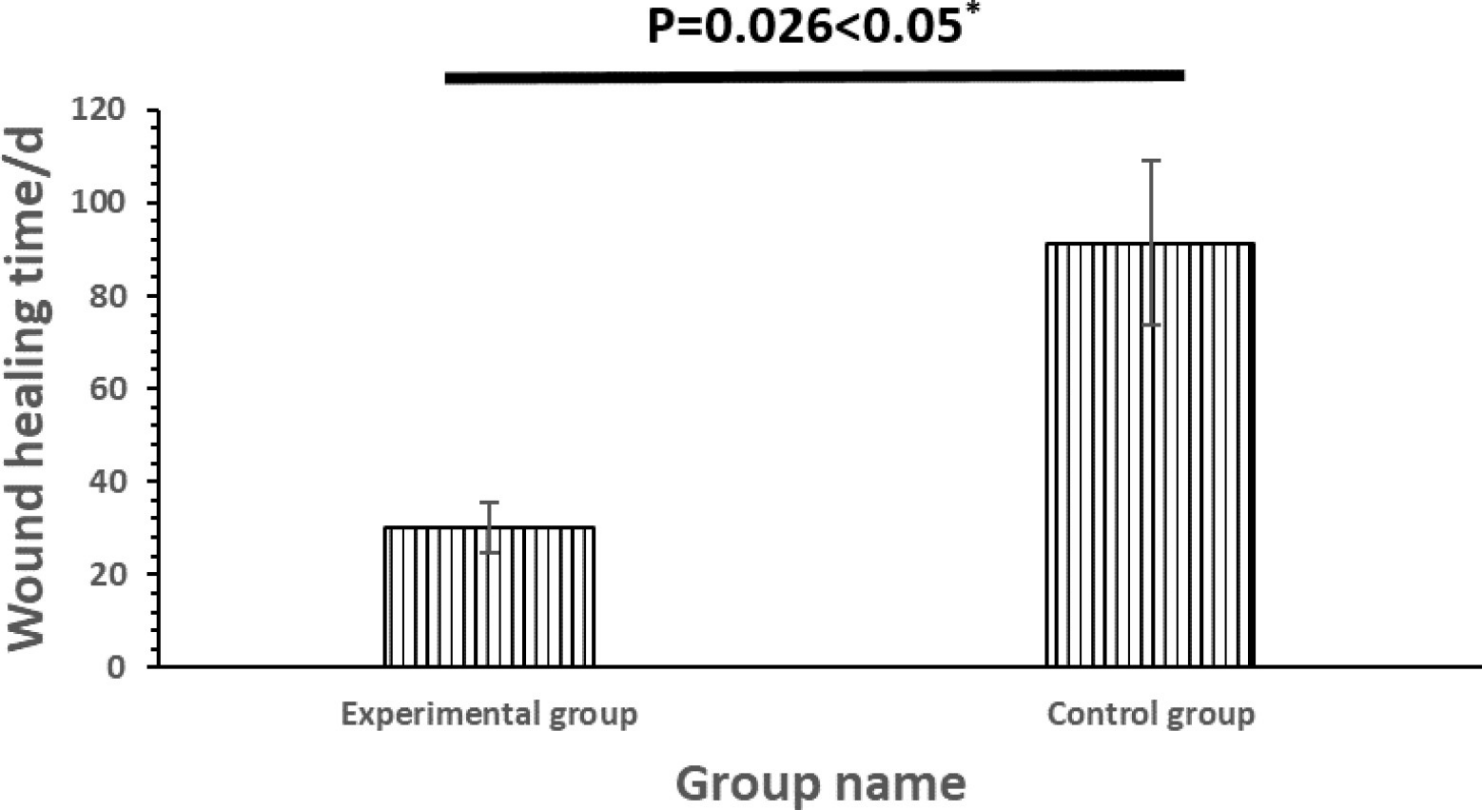
Comparison of wound healing time with cobra bite between two groups of patients.

As shown below in Fig 2, the wound healing time of the experimental group was significantly less than that of the control group (*p* < *0.05*). This indicates that the treatment plan of the experimental group (30.16 ± 5.37 days) was more effective than that of the control group (91.40 ± 17.81 days), shortening the wound healing time to approximately one month.

### 3.5 Images Comparison of the patient’s wound necrosis from the same site of the cobra bite between two study participants groups

**Fig 3:**
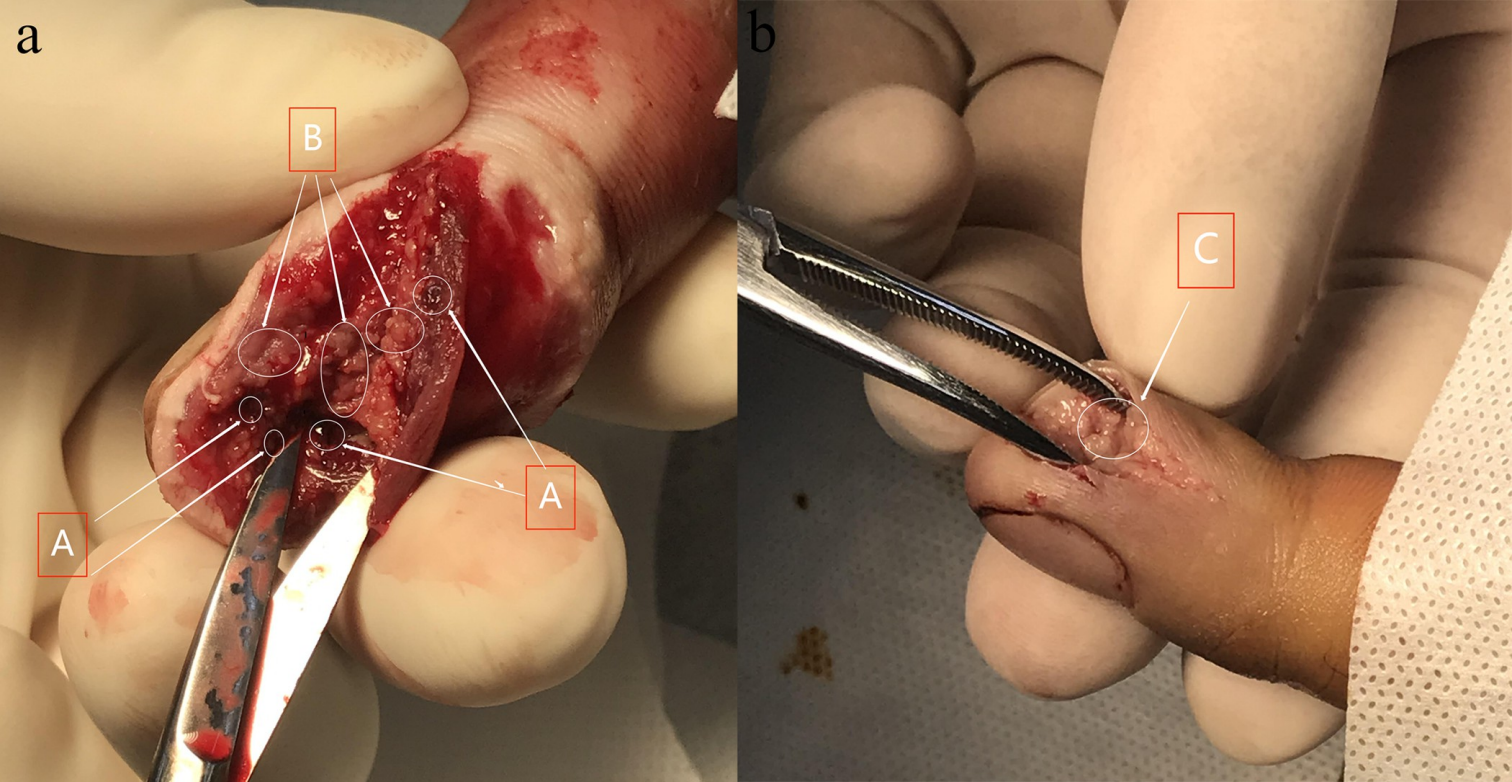
A. The wound surface on the third day of treatment in the control group; A shows small thrombus and B shows necrotic adipose tissue. C shows necrotic adipose tissue. Fig 3B. The wound surface on the third day of treatment in the experimental group; C shows necrotic adipose tissue.

Pictures of bites at the same site from the two groups of patients were randomly selected for comparison; the two figures above (Fig 3A and 3B) show the state of the wound after cleaning, without any surgical intervention. Fig 3A shows the wound surface on the third day of treatment of the control group with the Area A as small thrombus and Area B as necrotic adipose tissue. Meanwhile, Fig 3B shows the wound surface on the third day of treatment of the experimental group with Area C as necrotic adipose tissue. We found that the necrotic volume of the wound of the control group was significantly larger than that of the experimental group.

### 3.6 Pathological biopsy of wound healing tissue

**Fig 4:**
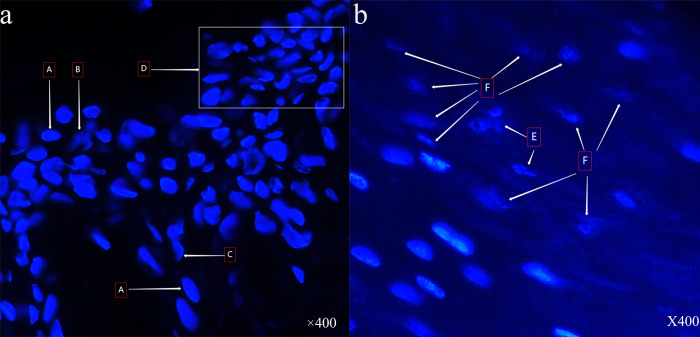
A. Pathological section staining of the patient’s wound in the control group (the microscopy magnification: 400X); A shows normal cells with nuclear staining morphology, B shows cells with fragmented nuclei, C shows cells with lobulated nucleus and D shows cells with irregularly polymorphic nuclei. Fig 4B. Pathological section staining of the patient’s wound in the experimental group (the microscopy magnification: 400X); E shows cells with lobulated nucleus and F shows the cells with blurred edges nucleus.

Hoechst 33258 staining was applied to the tissue sections. Visible pictures were then captured under a microscopic with 400× field of view. Fig 4A shows stained sections from the wound of patients in the experimental group that reveal that the nucleus density is high, and some of the nuclei are broken to form irregularly shaped vesicles; in Fig 4A, Area A shows normal cells with nuclear staining morphology, Area B shows cells with fragmented nuclei, Area C is cells with lobulated nucleus and Area D shows cells with irregularly polymorphic nuclei. Fig 4B shows the wound pathology of the control group patients, where most of the cells had sparse nuclear density, nuclear fragmentation, or nuclear consolidation, blurred and unclear nuclei edges, and extensive necrosis. Area E shows cells with lobulated nucleus and Area F shows the cells with blurred edges nucleus.

## 4. Discussion

Generally, cobra bites are characterized by high morbidity, and disability and often cause local tissue necrosis. To treat the local tissue necrosis caused by cytotoxins, “blocking therapy” is employed in the treatment of venomous snakebites, which is a normal method for the treatment of pain by applying a local anesthetic; after the sealing solution is added with chymotrypsin, it has a certain and clear effect against snakebites [24]. However, it is considered that anti-snake venom serum as a specific antibody appears to have a good and specific effect on snakebites and, compared with chymotrypsin, has more advantages in detoxification. In addition, from the results of this study, we also found that anti-snake venom blocking therapy showed superior clinical effects.

First, none of the patients developed serious complications requiring implants or amputations, which would cause disability. In addition, patients bitten by Chinese cobra usually develop delayed necrotic wounds [18,25]. With therapeutic intervention, the local necrotic volume reached a peak in three days. Over time, the cytotoxins degrade and are consumed, and fresh granulation tissue continues to grow out, thus reducing the necrotic volume of the wound. From our study, we found that both treatments showed good effects. However, the healing time of the wounds differed. The experimental group took approximately 30.16 days on average; while the control group took 91 days. Due to the different proportions of cobra venom in different regions, the main component of Chinese cobra venom is cytotoxin [15], therefore, the healing time of the wound bitten by Chinese cobra may be longer than that of *Naja kaouthia* and *Naja siamensis*. In addition, the peak necrotic volume of the wounds of the experimental group was significantly lower than that of the control group, indicating that the anti-snake venom closure treatment protocol is much more effective than the closure treatment protocol with chymotrypsin. Here, the closure treatment for venomous snakebites is carried out by injecting drugs that have a destructive effect on snake venom; a closed ring is then formed around the wound to prevent the spread of snake venom and prevent the damage of snake venom to local tissues. Commonly used drugs are chymotrypsin and pancreatic enzymes. It is important to note that patients bitten by Chinese cobra must use the corresponding anti-snake venom, not geographically diverse snake anti-snake venoms since antigenicities of anti-snake venoms have been found to vary from one species to another, whose titer may be very low or even ineffective [26–29].

There is no doubt that the blocking treatment of chymotrypsin has a certain effect on all kinds of snakebites. From Fig 3A, it can be seen that the wound necrotic volume of the control group was deep with severe necrosis of the adipose tissue (Area B part in Fig 3A) and dermis and the formation of tiny local thrombi (Area A part in Fig 3B), while that of the experimental group shown in Fig 3B was much smaller, and necrotic adipose tissue (Area C part in Fig 3B) was visible without the formation of tiny thrombi. It can be inferred that the formation of microscopic thrombi after cobra bites may also be an important factor in local tissue necrosis. The formation of small thrombus results in ischemia and hypoxia in local tissue cells, and also cannot make intravenous anti-snake venom reach the lesions. Once this pathological factor is formed, the use of anti-snake venom cannot reverse the situation. Therefore, it is critical to adopt anti-snake venom at the early stage after a cobra bite. The earlier anti-snake venom is used, the more effective it is. Although this study did not assess the prognosis of patients in terms of the timing of anti-snake venom administration, there is preliminary evidence from many similar studies that the early use of anti-snake venom is beneficial [4,12].

In addition, according to the pathological sections shown in Fig 4A and 4B, the nuclei of the experimental group and the control group were found to be lobulated (C, E) or broken (B). However, in the control group, a wide range of nuclei appeared in a state of dissolution with the nuclear boundary blurred (F). Under the same microscopic magnification, the nuclei in Fig 4B were sparse, which can be seen that the cell necrosis in the control group was more serious than that in the experimental group. From Fig 4A, the nuclei in Area D is irregular and polymorphous, which may be in the early stage of cell necrosis or the early and middle stage of cell apoptosis. The types of Cobra cytotoxin are diverse and the mechanism of action is complex. Through searching UniProt database, we found that there are 41 kinds of cytotoxins related to Chinese cobra, mainly divided into P-type and S-type. The P-type is more toxic such as CTX9 and CTX10 while CTX2, CTX7, and CTX8 belong to the S-type [30]. Generally, cytotoxin will interact with the cell membrane to dehydrate the membrane surface and destroy the lipid bilayer structure [31], which can form dimers on the surface of the cell membrane, and then form oligomeric complexes [32]. Finally, it would result in the formation of pores in the membrane lipid bilayer, the leakage of cytoplasm, and the breakdown and dissolution of cells [33]. Actually, cytotoxin not only has the activity of dissolving cells, but also can induce apoptosis and further aggravate wound necrosis: for example, CTX1 and CTX2 can induce apoptosis through the lysosomal pathway and the release of cathepsin to the cytoplasm. Furthermore, a transition from apoptosis to necroptosis can occur with the toxin concentration increased [34–36]. However, our study could conclude that no matter what mechanism of cytotoxin leads to cell death, applying anti-snake venom blocking therapy is appropriate.

However, there may be other factors involved in local tissue necrosis, and the wound may be complicated due to variety of bacterial infections [37–41]. Some studies have reported that 27.17% of patients with cobra bites had wound infections [38]. Therefore, cobra bites should immediately be cleaned, debrided, and dressed as soon as possible, and given antibiotics to reduce the incidence of infection [42,43]. Additionally, some reports have shown that wound necrosis is related to the patient’s treatment time, the concentration of toxin, and the dose of anti-snake venom; the less toxin secreted by the cobra, the earlier the patient visits the medical practitioner, and the higher the dose of anti-snake venom used, the lower the probability of wound necrosis [44,45]. Pharmacokinetics indicated that higher doses of anti-snake venom administered for cobra envenoming facilitate the neutralization of venom antigens [46], but still can’t completely neutralize the residual venom depot at the site of wound [47], which may remain active and slowly lead to local tissue necrosis. Therefore, in a case of local tissue destruction caused by cobra venom, a higher dose of anti-snake venom is required. However, local blocking therapy with anti-snake venom can meet the treatment requirements, such as a high concentration dose for local tissues. Therefore, a stock solution of anti-snake venom for local closure treatment should be used. Anti-snake venom does not need to be diluted with normal saline, and the higher the concentration of anti-snake venom, the more effective and beneficial the treatment will be for the patients.

Based on the above experimental results, the clinical efficacy of anti-snake venom blocking therapy is much better than the blocking therapy of chymotrypsin; the difference in clinical efficacy is mainly determined by the different pharmacological properties of chymotrypsin and anti-snake venom. As shown in Table 3, in the control group: the wound surface significantly increased from the first day (46.20 ± 28.22 mm^3^) to the third day (1674.44 ± 972.35 mm^3^), which indicated that chymotrypsin may have a relatively weak destructive effect on snake venom protein, although it is a proteolytic enzyme; while in the experimental group: the increment of wound surface from the first day (59.84 ± 36.68 mm^3^) to the third day (71.16 ± 41.56 mm^3^) was relatively small, which can be seen that the anti-snake venom serum has a strong neutralizing ability against snake venom, because it is a specific antibody. Therefore, it may be the high efficiency, specificity and specificity of antibodies that determine the better clinical efficacy of anti-snake venom blocking therapy. However, chymotrypsin has some effects in promoting wound repair. Anti-snake venom serum plays a role in the early stage of wound necrosis to neutralize local toxins and prevent wound expansion. But it may not be appropriate to combine the two kinds of blocking therapy, which may interact to reduce the efficacy.

The statistics of adverse events in this study showed that only two of the 50 patients had allergic reactions, and the incidence of allergy was 4%. According to the current research, the allergic rates of anti-snake venom serum reported in different countries are different with the incidence ranging from 2–50% [48–50]. It can be seen that the adverse reactions of our study are within a normal and reasonable range, which proves that the anti-snake venom serum blocking treatment scheme can minimize the local tissue necrosis to the greatest extent, reduce the disability rate, and bring huge benefits to patients. Furthermore, this treatment plan is also applicable to wound necrosis caused by other snake toxins, such as tissue necrosis caused by elapidae and viper family. Therefore, future research should not only be limited to the field of treating cobra bites but also give full attention to the toxicological properties of cobra toxins for the prevention and treatment of certain diseases, which currently seem to have a broad scope of application. The innovation of this study is the use of anti-snake venom as a local occlusion therapy, as opposed to the traditional treatment method of anti-snake venom via intravenous injection and local treatment with chymotrypsin.

## 5. Conclusion

Snakebite is a common clinical emergency, and local tissue necrosis caused by cobra bites is a difficult focus of clinical treatment. This study improves the traditional chymotrypsin closure treatment method by using anti-snake venom for local closure treatment of snakebite wounds so that the concentration of anti-snake venom in local tissues is higher than that in human serum, which can better neutralize the venom, stop the spread of the venom, and reduce tissue degeneration and necrosis. This treatment plan has little side effects and will not increase the allergic reaction of anti-snake venom serum. This new treatment protocol has much better clinical efficacy, can obtain more benefit for patients, and is thus worth promoting in clinical treatment.

## Supporting information

S1 CONSORT Checklist CONSORT 2010 checklist of information to include when reporting a randomized trial. (DOC)

S1 Fig Therapeutic schedule of local tissue necrosis caused by Chinese cobra (*Naja atra*) bites. (TIF)
