# Supplementary figures and images for: Clinical study of anti-snake venom blockade in the treatment of local tissue necrosis caused by Chinese cobra (Naja atra) bites
Source: PLoS Negl Trop Dis. 2022 Dec 16;16(12):e0010997. doi: 10.1371/journal.pntd.0010997 (PMC9803274; doi:10.1371/journal.pntd.0010997)

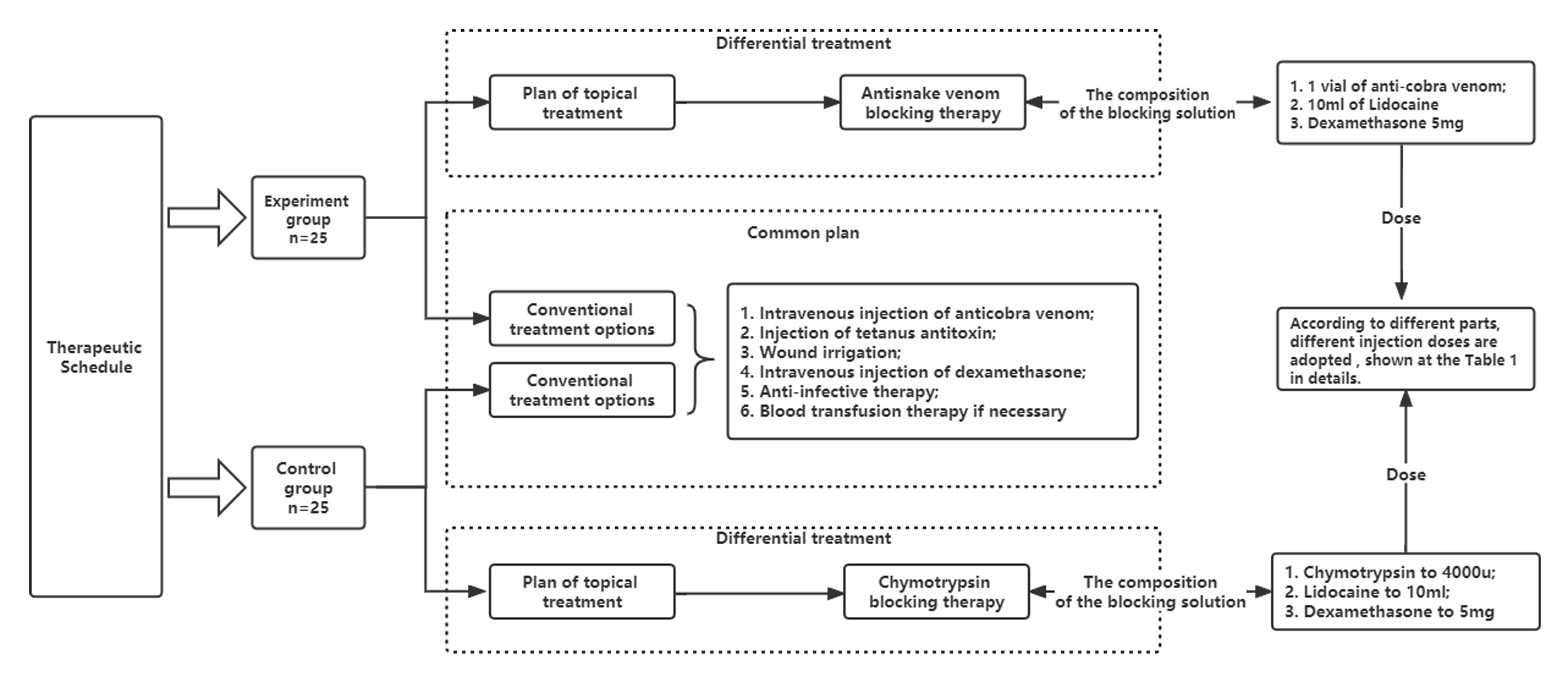

Supplement: S1 Fig — (TIF) [file pntd.0010997.s002.tif]
